# Environment friendly pesticide formulation by adding certain adjuvants and their biological performance against *Sitophilus oryzae *(L.)

**DOI:** 10.1038/s41598-024-83922-2

**Published:** 2025-01-07

**Authors:** Saad A. S. A., Ahmed M. A. Kordy, Marwa I. Mackled, Amira E. Ahmed, Shimaa S. Abd El-Naby

**Affiliations:** 1 Plant Protection Department, Faculty of Agriculture (Saba Basha), Alexandria University, 21531 Alexandria, Egypt; 2 Department of Stored Product Pests, Plant Protection Institute, Agriculture Research Center (ARC), Sabahia, Alexandria Egypt; 3 Pesticide Formulation Research Department, Central Agriculture Pesticides Laboratory, Agricultural Research Center, Alexandria, Egypt

**Keywords:** Adjuvant, Physicochemical properties, Nanoemulsion, Sesame, Clove, Cinnamon, *S. oryzae*, Kinetic stability, Biological techniques, Zoology, Nanoscience and technology

## Abstract

Formulation and adjuvant technologies can facilitate the use of insecticides that have higher biological efficiency application features. Safety, physicochemical properties by increasing consumer demand for safe food and enhancing operator safety. The aim of this current work was to develop a green efficient, and stable pesticide formulation. Therefore, certain nano emulsions with and without Adjuvants Calcium Alkyl Benzene Sulphonate (Atlox 4838B), and non-ionic surfactant based on trisiloxane ethoxylate (ARGAL), were testing against *Sitophilus oryzae* (Coleoptera: Curculionidae). Certain analytical techniques were used for determining the characterization of the nano emulsions (Sesame, Clove, and Cinnamon). Results showed that all formulations were penal, achieving nanometric size for all compounds. Scanning Electron Microscopy (SEM) micrographs revealed spherical or quasi-spherical morphologies for the tested nanoemulsion formulation nanodroplets. Furthermore, dynamic light scattering (DLS) showed that the particle size of the formulation with the adjuvants showed a slight increase in the droplet size compared to the formulations without adjuvants. In comparison to the tested nanoemulsions with adjuvants, the viscosity of the nanoemulsions without adjuvants was lower. All studied formulations, both with and without adjuvant, showed an acidic to slightly acidic pH, except for sesame (NE) with AtloxTM 4838B, which showed a neutral pH, and they were kinetically stable with no phase separation, creaming, or crystallization. Furthermore, supporting the stability of these nanoemulsion particles was the absence of a separation phase following centrifugation, freeze–thaw cycles, and heating–cooling cycles. Findings proved that ARGAL and Atlox 4838B adjuvant stabilized NE by increasing Brownian motion, weakening the attractive forces with smaller droplets, increasing the value of zeta potential and polydispersive index (< 0.6), and decreasing surface tension. The bioassay technique using film residue to estimate LC50 values on *S. oryzae* adults indicate that Clove, Sesame, and Cinnamon nano emulsions with Atolx adjuvants were the most effective against *S. oryzae* adults under laboratory conditions, where the LC50 Values are 0.022, 0.032 and 0.035 µL/cm^2^ respectively after 27 h, or exposure time. Clove, Cinnamon, and Sesame nanoemulsion (NE) with 0.01% (w/w) adjuvant exhibited remarkable insecticidal activity against *S. oryzae* L., of 100, 100 and 97.5% respectively by in vitro assay.

## Introduction

Today, agrochemical formulation development technology has become a well -established field that not only offers formulators significant added value, but also presents pesticide users with attractive products by enhancing operator safety^1^. Current agrochemical discovery faces major challenges in a rapidly evolving market^2^. These challenges including agrochemical resistance and their management, pest shifts^3^ changes in public perception of agricultural technology, the increasing cost of research and progress^4^ and evolving regulatory requirements^2^. Lowering dosage rates and crop pesticide waste and ultimately improving environmental quality^5,6^. Plant essential and natural oils can be employed as biopesticides as an alternative strategy for crop productivity and integrated pest control^7^. Cloves (*Syzygium aromaticum*) belong to the Myrtaceae plant family. It is an aromatic flower that grows in Sri Lanka, China, Indonesia, and Madagascar. According to various sources, clove essential oil includes a large quantity of phenolic compounds Eugenol and Eugenol acetate^8^, which have a variety of biological activities, including antibacterial, antifungal, insecticidal, and antioxidant characteristics^9^. Cinnamomum zeylanicum is a Lauraceae plant that grows in India, Sri Lanka, Indochina, and Madagascar^10^. Jamaica and Brazil.^11^. Cinnamaldehyde, is a naturally occurring chemical found in the bark of cinnamon trees and other members of the Cinnamomum genus. Cinnamaldehyde contains about 90% of the essential oil extracted from bark. Cinnamon oil also includes many chemical components (including cinnamic acid, coumaric acid, cinnamyl alcohol, and eugenol) with antioxidant and antibacterial properties^12^. Furthermore, insecticidal and repellant properties^13^.

Sesame (*Sesamum indicum* L.) is one of the oldest oil crops used for human consumption in the Pedaliaceae family^14^, mostly produced in India, Sudan, Myanmar, China, and Tanzania. Sesame oil is high in unsaturated fatty acids (linoleic acid, palmitic acid, stearic acid, oleic acid, arachidic acid, palmitoleic acid, linolenic acid, lignoceric acid, behenic acid, myristic acid, caproic acid, and margaric acid)^15,16^. Sesame is not only high in nutrients, but it also includes several key functional components such as sesamin, sesamol, sesamolin, sesaminol, sesamolin phenol, and other lignan-like active compounds^17^. Sesamin is a fungicide and insecticide that may be used in conjunction with pyrethroid insecticides^18^. The difficulty with using essential oils as insecticides is that they show poor solubility in water, high volatility, and sensitivity to environmental stresses^19^. In order to address this issue, essential oils can be formulated into nanoemulsions, which lessen the bioactive compounds’ volatility, hydrophobicity, and reactivity^20^. Nanoemulsion (NE) is an oil-in-water emulsion containing small, spherical oil droplets that are less than 200 nm in size^21^. It lowers the hydrophobicity, volatility, and reactivity of bioactive chemicals found in essential oils^20^. Furthermore, it improves the contact area between the components, spreading the oil across a broader surface and increasing biological activity. On the other hand, nanoemulsions are thermodynamically unstable, implying that nanodroplets might destabilize in numerous ways as they age. Coalescence and Ostwald ripening are well-known destabilizing processes in nanoemulsions^22,23^. As a result, there is an urgent need to identify effective adjutants that may stabilize the active components of most desirable nanoemulsion pesticides and provide increasing bio-efficacy and stability for a longer period. Additives have the potential to both improve the physical properties of pesticide formulations that have been pre-diluted as well as their biological efficacy. So, selecting the proper adjuvant is critical for establishing a stable nanoemulsion because of a high repulsive force that inhibits flocculation and coalescence between nanodroplets^24^.

Organosilicone and Alkyl Benzene Sulfonates adjuvants are compounds that are added to pesticides and other agricultural chemicals to increase droplet spreading and wetting. They also lower the spray solution’s surface tension, which increases pesticide penetration and results in a uniform distribution of the active ingredient on the plant surface^25,26^. Additives have the potential to both improve the physical properties of pesticide formulations that have been pre-diluted as well as their biological efficacy^27^. For instance^28^, reported that neem nanoemulsion with botanical adjuvant provided long-term storage stability as well as better adhesiveness and wetting with lower surface tension, as well as, Neem NE with adjuvant showed remarkable insecticidal effectiveness against whitefly. Furthermore^29^, found that adding adjuvants to olibanum NE improved stability and lowered surface tension and contact angle more effectively than olibanum NE alone. Additionally, the olibanum NE, with and without adjuvants, was the most effective chemical against the second instar larvae of the spiny bollworm (*Earias insulana*). Also^30^, demonstrated that the solid lipid nanoparticles (SLNPs) of citronella oil combined with three adjuvants increased the toxicity and mortality percentage of *Spodoptera littoralis* larvae, as well as stability, more than the nano formulation alone, furthermore^31^, evaluated the insecticidal efficacies and chemical compositions of some essential oil against the rice weevils, (*S. oryzae* L.). Also, the chemical pesticides such as lambda-cyhalothrin, Abamectin, Emamectin benzoate, Imidaclopride, and Oxamyl were detected to be more effective and longer-lasting effects when PEG 600 di-oleate adjuvants were added^32,33^. Adjuvants improved the efficacy of lambda-cyhalothrin formulations, according to^34,35^. Adjuvants or other inert chemicals can be added to neem NE to increase its biological activity^36^. Consequently, the number of treatments each season and the pace at which pesticides are applied can both be decreased by using adjuvants. As far as we are aware, no literature exists on the insecticidal properties of an improved formulation of sesame, clove, and cinnamon NE with different adjuvants against *S. oryzae*. So the purpose of this study was to specify the effect of two additives on increasing the effectiveness of the prepared nanoemulsion formulation of sesame, clove, and cinnamon oil against *S. oryzae*, maintaining or improving the physicochemical properties of the formulation, and aiding in the transition from preventative, high-dose applications to low-dose. Thus, this study could provide technical support for the development and utilization of efficient formulation.

## Materials and methods

Clove (*S. aromaticum*), cinnamon (*C. zeylanicum*), and sesame (*S. indicum*) oils were supplied by the Production of Medicinal Plants and Extracts Unit (National Research Centre, Egypt). Tween 80 was acquired from Scientific Distributors located in Egypt. ARGAL (Silwet) is a non-ionic surfactant based on trisiloxane ethoxylate, an organosilicone adjuvant supplied by Shoura Chemicals Company (Egypt). Calcium Alkyl Benzene Sulphonate (Atlox 4838B) is an anionic surfactant. Chemical Group Alkyl Benzene Sulfonates adjuvant was supplied by Target for Chemicals Industry & Trade distributer (Egypt).

### Nanoemulsion preparation

NE formulations were developed by the high-energy method, as previously described by^37^**.** NE were created in two processes, the initial stage included creating emulsions by mixing three key components oil, water, and surfactant, were mixed using a magnetic stirrer that revolved at 500 rpm for 45 min. The emulsions were then transformed into nanoemulsions using a 20 kHz Probe Sonicator (BANDELIN Sonopuls, Germany). After that, the prepared quantity divides into two quantities: the first one, in which add 0.01% (w/w) of both adjuvants to the prepared nano formulations, and the other quantity, without adjuvant.

### Characterization and physico-chemical properties of nanoformulations

#### Particle size analysis

The mean particle size (z-average, z-ave) and polydispersity index (PI) were determined using a Zetasizer Nano ZS (Malvern-UK, 4700 model, Germany). Photon correlation spectroscopy produces the hydrodynamic diameter, which is an intensity-weighted average diameter of the bulk population. PI represents the width of the particle size distribution. To achieve the desired scattering intensity, samples were diluted with water prior to analysis. For this, 10 µL of material was mixed with about 10 µL of water.

#### Surface morphological analysis.

The morphological study of the tested formulations was carried out using JSM-IT200 (JJEOL, Japan) microscopes and scanning electron microscopy (SEM) methods, as described by^38^. Briefly, the samples were diluted in distilled water at 1:300 (v/v)., a 60 μL aliquot was placed on a smooth carbon tape that was attached to a stub surface. It was then contrasted with 2% OsO4 vapor for 30 min and allowed to dry at room temperature for 24 h. Following full droplet drying, the samples were metalized with gold using a splatter cutter, and then images were taken.

#### Kinetic stability assessment

The approach previously described by^37^ was utilized to measure kinetic stability. Sesame, Clove, and Cinnamon nano emulsions with and without adjuvant were stored for 48 h at different temperatures (0°C and 45°C) to examine their thermodynamic stability. For fifteen minutes, the nanoemulsion formulations were centrifuged at 4000 rpm. Freezing–thawing experiments were also used to verify the kinetic stability of the tested nano emulsions. These tests were conducted for a minimum of 24 h at two distinct temperatures (− 21°C and 25°C).

#### Zeta potential analysis

Electrophoretic mobility was measured using a Malvern Zetasizer Nano ZS (Malvern Instruments, Malvern-UK, 4,700 models, Germany) to calculate the zeta potential. Samples were prepared as reported by^39^ and distributed in a cuvette with 20 μL of deionized water.

#### PH Measurement

The pH value of NEs with and without adjuvant was determined using a pH meter (Adwa—AD8000) by dipping the electrode in the sample and leaving it at room temperature for 5 min without stirring to let the pH value stabilize^28^.

#### Viscosity measurement

The dynamic viscosity of NEs with or without adjuvants was determined using a Brookfield DV II + PRO" digital viscometer (Brookfield, USA). The viscosity (cPs) of each formulation was instantly assessed using a viscometer^40^.

#### Surface tension

The tested nano formulations surface tension was measured in triplicate at room temperature using a Sigma Force Tensiometer 700 USA^41^.

### Creaming index

The creaming index was determined using the previously stated methods by^42,43^. Briefly, 10 gm samples of nanoemulsion with and without adjuvant were put in sealed, tight containers and stored at 25 °C for seven days. Because oil droplets have a lower density than water, they rise to the surface during storage. Height measurements were taken for both the depleted bottom layer (HD) and the entire emulsifier. The creaming index was calculated using the following formula:

$$\text{Creaming Index}=\text{ HD}/\text{HE} \times 100$$

### Insect rearing

Rice weevils**,** *S. oryzae* (L.) were raised on previously sterilized rice and kept in a pesticide-free environment for almost five years at the Plant Protection Research Institute at Egypt’s Stored Grains and Product Pests Research Department^44,45^. All studies, including insect rearing, were conducted at 26 ± 1°C and 65 ± 5% R.H. Adults used in trials were two weeks after eclosion, as described by^44,46^.

### Bioassays examinations

#### Acute toxicity of tested nanoformulations

The direct toxicity effect of the sesame, clove, and cinnamon nanoemulsion with and without adjuvant on adults of *S. oryzae* was assessed using a direct contact assay^47–49^. In a glass Petri dish (9 cm diam.) 2ml acetone solvent and the different concentrations (2, 4, 6, 8, and 10µl (v/v) of the tested nanoemulsion with and without adjuvant were applied using a micropipette and spread uniformly along the whole surface of the Petri dishes. The solvent was left to evaporate then, twenty adults of *S. oryzae* were placed in separate petri dishes. The dishes that simply contained solvent were utilized as a control treatment. After 24, 48, and 72 h of treatment, the number of dead insects was counted, and mortality percentages were computed.

### Statistical analysis

Probit analyses were calculated using a computerized software application (Ld-p line). Copyright by Ehab, M. Bakr, Plant Research Institute, ARC, Giza, Egyp., to obtain LC50 and LC90 values of the tested nanoemulsions with and without adjuvant according to^50^ and mortality was expressed as mean ± SD.

## Results

### Physico-chemical properties characterization of nanoformulations with and without adjuvant

#### Droplet size and polydispersity index (PDI)

**Fig. 1:**
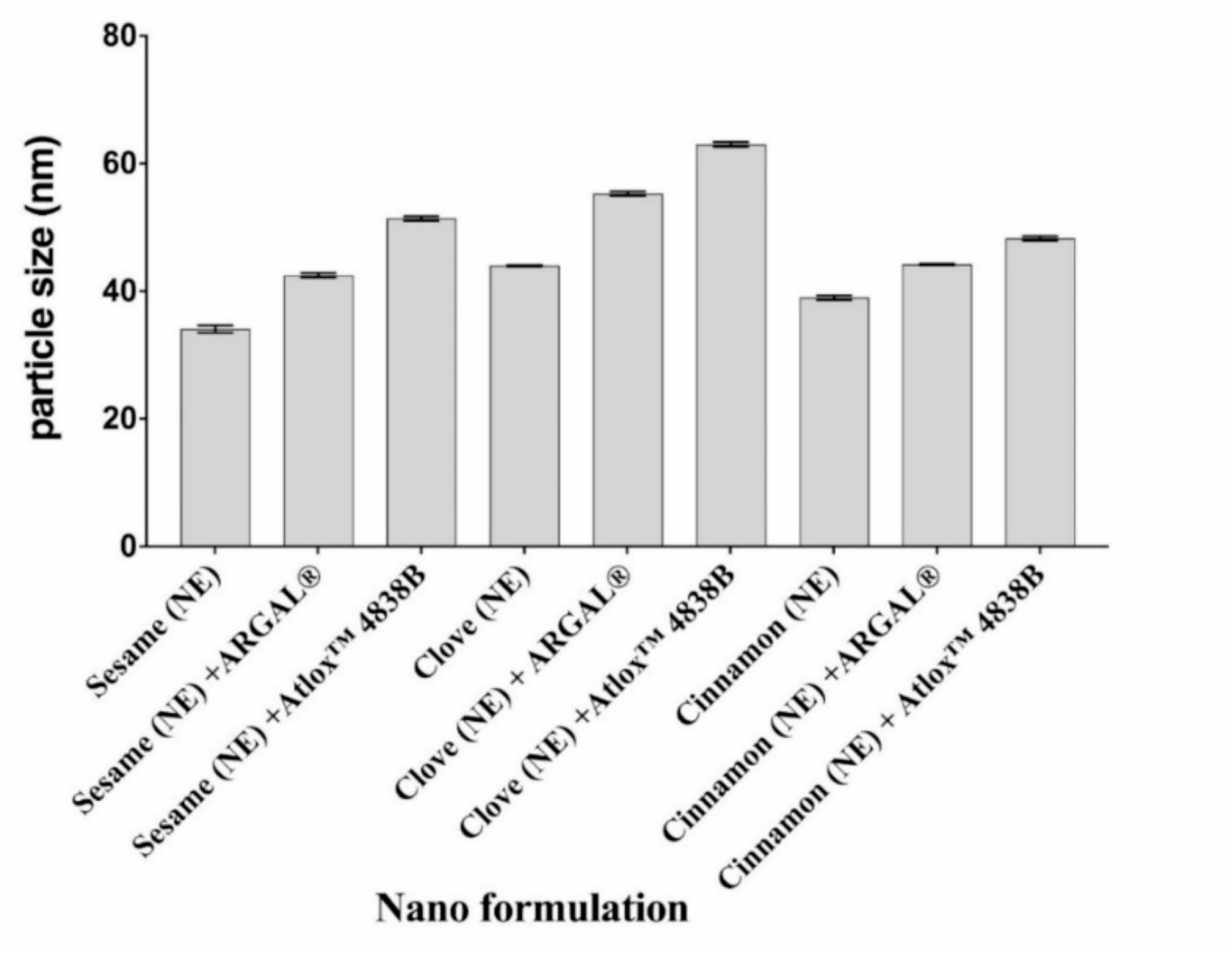
Determination of droplet size distribution using dynamic light scattering (DLS) of Sesame, Clove, and Cinnamon nanoemulsion with and without adjuvant. Bar charts are generated using data from four biological replications with two technical replicates per replication. Each bar represents the mean, and the error bar indicates the standard deviation (± SD). This figure was drawn with GraphPad Prism 8 (9.4.1, (458) Serial number: GPS-2567891- 8A130A8A228).

**Fig. 2:**
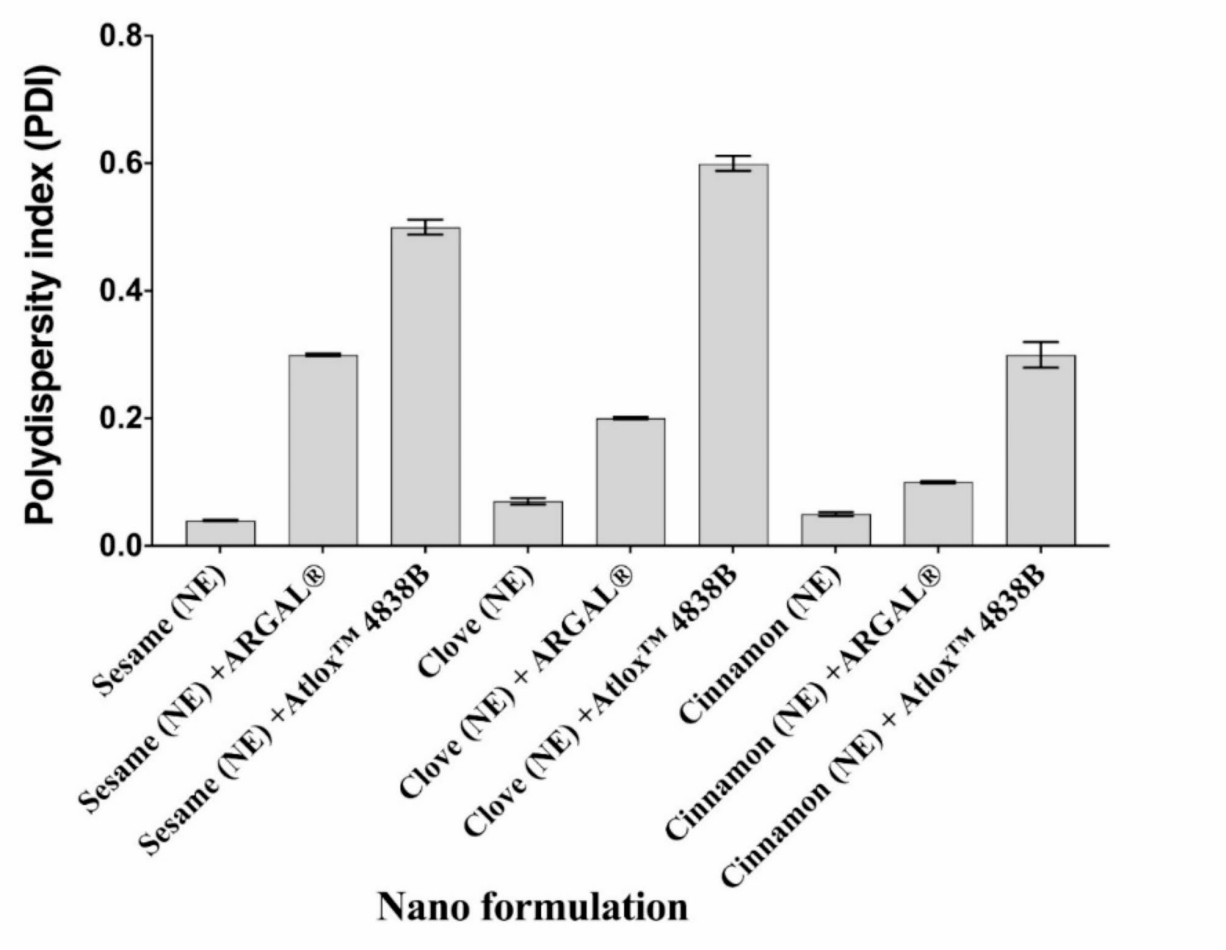
Polydispersity index (PDI) of sesame, clove, and cinnamon nanoemulsions with and without adjuvant. Bar charts are generated using data from four biological replications with two technical replicates per replication. Each bar represents the mean, and the error bar indicates the standard deviation (± SD). This figure was drawn with GraphPad Prism 8 (9.4.1, (458) Serial number: GPS-2567891- 8A130A8A228).

Droplet size and PDI are presented in Table 1 and Fig. 1, shows the droplet size of the prepared nano emulsions with and without adjuvant by a dynamic light scattering (DLS). The results reflected that nanoemulsion formulation recorded droplet size of 34.09 nm for Sesame (NE), 42.5 nm for Sesame (NE) with ARGAL, and 51.4 nm for Sesame (NE) with Atlox 4838B. While, the droplet size of Clove (NE) was 42.5 nm, Clove (NE) with ARGAL was 55.3nm and Clove (NE) with Atlox 4838B was 62.5nm. However, Cinnamon (NE) was 37.8 nm, Cinnamon (NE) with ARGAL was 44.2nm and Cinnamon (NE) with Atlox 4838B was 48.3nm. This result demonstrated that preparation in the nanometric size for all compounds was successful. Nanoemulsion formulation Sesame (NE) and Cinnamon (NE) recorded the lowest value of droplet diameter size (34.09 and 37.8nm, respectively) compared to NEs Clove (NE) was 42.5 nm. The polydispersity index, a dimensionless measure of the broadness of the size distribution obtained from cumulants analysis, was found to be 0.04, 0.07, and 0.05 for Sesame, Clove, and Cinnamon respectively, (Table 1 and Fig. 2).

**Table 1:** Physico-chemical properties of Sesame, Clove, and Cinnamon nanoemulsions with and without adjuvant.

Nano formulation	Stability indices						Surface tension	Particle size (nm)	PDI	Viscosity (cPs)
	Creaming index	Centrifugation test	Freeze–Thaw cycles	pH value	Heating–cooling cycle	Zeta potential (mV)				
Sesame (NE)	Stable	Stable	Stable	6.96	Stable	-31.3	50.1	34.09	0.04	35.3
Sesame (NE) + ARGAL	Stable	Stable	Stable	6.63	Stable	-35.2	45.3	42.5	0.3	38.5
Sesame (NE) + Atlox 4838B	Stable	Stable	Stable	7.12	Stable	-39.5	40.2	51.4	0.5	40.2
Clove (NE)	Stable	Stable	Stable	5.63	Stable	-29.2	44.9	42.5	0.07	33.6
Clove (NE) + ARGAL	Stable	Stable	Stable	5.52	Stable	-33.5	37.2	55.3	0.2	35.8
Clove (NE) + Atlox 4838B	Stable	Stable	Stable	6.09	Stable	-37.8	30.9	62.5	0.6	39.1
Cinnamon (NE)	Stable	Stable	Stable	4.53	Stable	-34.4	43.9	37.8	0.05	36.9
Cinnamon (NE) + ARGAL	Stable	Stable	Stable	4.27	Stable	-38.5	35.1	44.2	0.1	39.3
Cinnamon (NE) + Atlox 4838B	Stable	Stable	Stable	5.72	Stable	-40.7	29.3	48.3	0.3	38.8

While the tested nano emulsions with adjuvant recorded, 0.3, 0.5, 0.2, 0.6, 0.1 and 0.3 for Sesame (NE) with ARGAL, Sesame (NE) with Atlox 4838B, Clove (NE) with ARGAL, Clove (NE) with Atlox 4838B, Cinnamon (NE) with ARGAL, and Cinnamon (NE) with Atlox 4838B. These results indicated that there was a relatively narrow size distribution and homogeneity and uniformity of the formulations. A sample is almost monodispersed if the PDI is less than 0.08, and the midrange of PDI values is 0.08–0.70.

#### Surface morphological analysis

**Fig. 3:**
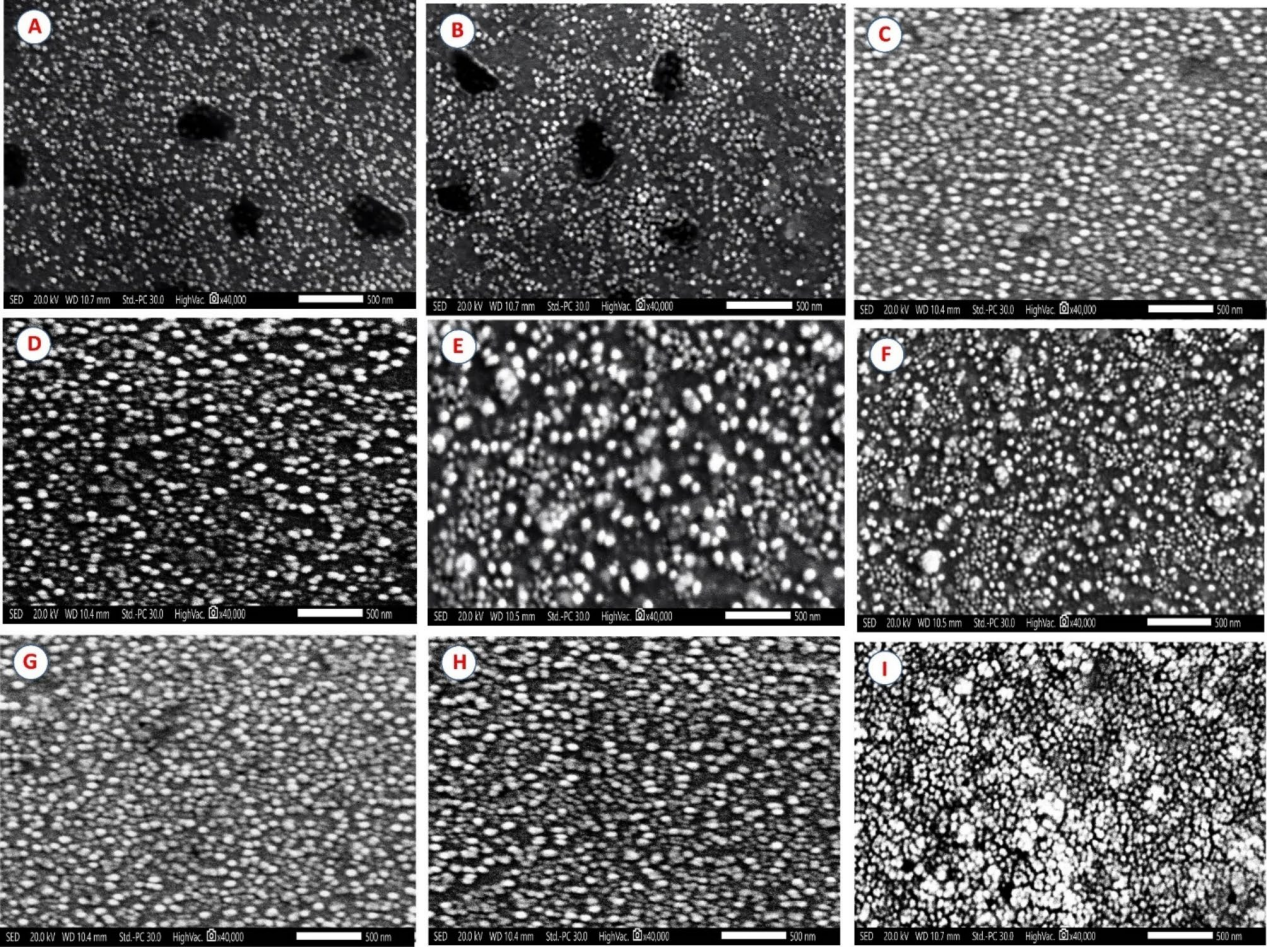
SEM images for Cinnamon (NE) (**A**), Cinnamon (NE) + ARGAL (**B**), Cinnamon (NE) + Atlox 4838B (**C**), Clove (NE) (**D**), Clove (NE) + ARGAL (**E**), Clove (NE) + Atlox 4838B (**F**), Sesame (NE) (**G**), Sesame (NE) + ARGAL (**H**), and Sesame (NE) + Atlox 4838B (**I**).

Scanning electron microscopy (SEM) was used to provide ultrastructural studies of the tested nanoemulsion formulation, providing further information on the nanosystem’s morphological features. Representative ultramicrographs (Fig. 3) obtained with this approach revealed spherical or quasi-spherical morphologies for the tested nanoemulsion formulation nanodroplets.

#### Viscosity measurement

The appropriate viscosity of a formulation is required for long-term storage without sedimentation, complete transfer from container to spray tank, homogenous distribution for spray solution and adhesion, and reduced run-off from target surfaces. Further reduces the nanoemulsion’s rate of aggregation during storage. ARGAL and Atlox 4838B adjuvants were used to optimize the viscosity of Sesame, Clove, and Cinnamon nanoemulsions, resulting in a stable formulation. As seen in Table 1 the viscosity of the studied nanoemulsions alone ranged between 35.3 and 36.9 cPs, while the viscosity of the tested nanoemulsions with adjuvants rose in the range from 35.8 to 40.2 cPs.

#### Surface tension

The surface tension value for Sesame, Clove, and Cinnamon nanoemulsion with and without adjuvant as reported in Table 1 were 50.1, 44.9 and 43.9 dyne/cm for Sesame, Clove, and Cinnamon nanoemulsions alone respectively, which was much lower than the surface tension of water (72 dyne/cm). The adjuvant addition was found to reduce the surface tension of the Sesame, Clove, and Cinnamon nanoemulsions. When a formulation’s surface tension decreases, the area of contact increases, that causes an increase around contact between the sprayed surface and the spray, in addation improving the wetting and spreading properties.

#### Influence of adjuvants on Sesame, Clove, and Cinnamon nanoemulsions stability

**Fig. 4:**
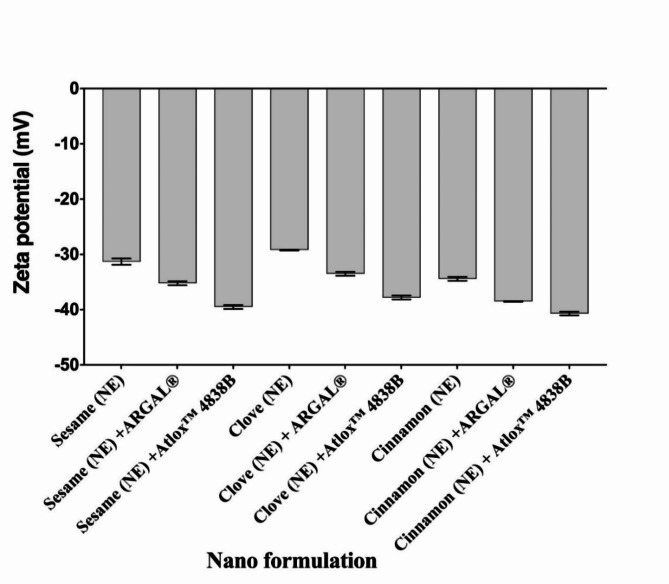
Zeta potential measurement of sesame, clove, and cinnamon nanoemulsion with and without adjuvant using Malvern Zetasizer Nano ZS. Bar charts are generated using data from four biological replications with two technical replicates per replication. Each bar represents the mean, and the error bar indicates the standard deviation (± SD). This figure was drawn with GraphPad Prism 8 (9.4.1, (458) Serial number: GPS-2567891- 8A130A8A228).

Stability is a critical characteristic in nanoemulsion systems. Because nanoemulsion systems have a large surface area and small droplet sizes. Nanoemulsions’ tiny droplet size gives them stability against creaming or sedimentation due to Brownian motion, and as a result, the diffusion rate is higher than the sedimentation rate generated by gravity force (Table 1). Nanoemulsions’ stability was examined under various temperatures. The tested nanoemulsion with and without adjuvant appearance was observed formulations’ pH was in a range (4.2–6.9), exhibited an acidic to slightly acidic except sesame (NE) with Atlox 4838B recorded (7.12). Measurements of the zeta potential in Fig. 4 showed that the nanoemulsion is homogenous. Data in Table 1 and Fig. 4 illustrated the zeta potentials for Sesame, Clove, and Cinnamon nanoemulsions with and without adjuvants within the zone of negative zeta potential. The nanoformulation of Sesame, Clove, and Cinnamon nanoemulsion alone has a zeta potential of − 31.3, − 29.2, and − 34.4 mV, respectively.

While adding adjuvant increased the value of zeta potential, as shown in Table 1 and Fig. 4. Particles having zeta potentials greater Values above ± 30 mV are regarded as stable. Because the electric charge of the droplets is large, it is reasonable to suppose that repulsive interactions between droplets exist in the nanoemulsion. A negative zeta potential value in a dispersed emulsion inhibits coagulation and coalescence by establishing repulsive forces between droplets that are greater than their attraction forces. The findings showed that the varied values and consistent distribution of the nanoformulation of sesame, clove, and cinnamon nanoemulsions with and without adjuvant indicated their stability. These results demonstrated that stability of the Sesame, Clove, and Cinnamon nanoemulsions due to the constant distribution and varying values of the nanoformulation with adjuvant.

### Mortality bioassay (contact toxicity)

The current findings showed that adding Calcium Alkyl Benzene Sulphonate adjuvant to Clove, Cinnamon, and Sesame nanoemulsion performed a high performance against the rice weevils, *S. oryzae* after a 27-h exposure period. The LC_50_ values of Clove, Sesame, and Cinnamon nanoemulsion with Atlox 4838B were 0.022, 0.032, and 0.035 µl/cm^2^, respectively, followed by ARGAL adding Cinnamon, Sesame, and Clove nanoemulsion with an LC_50_ value of 0.031, 0.055, and 0.056 µl/cm^2^, respectively. While the LC_50_ values of Clove, Cinnamon, and Sesame nanoemulsion were 0.052, 0.064, and 0.071 µl/cm^2^, respectively, following a treatment of 72 h (Table 2). Furthermore, the order of tested nanoemulsions with and without adding adjuvant efficacy against adults of the rice weevil based on LC_90_ values also exhibited a similar tendency of almost LC_50_ values. The LC_90_ values of Clove, Cinnamon, and Sesame nanoemulsion with Atlox 4838B were 0.111, 0.112, and 0.138µl/cm^2^, respectively, followed by ARGAL adding Cinnamon, Sesame, and Clove nanoemulsion with an LC_90_ value of 0.130, 0.213, and 0.326 µl/cm^2^, respectively, while the LC_90_ values of Cinnamon, Clove, and Sesame nanoemulsion were 0.295, 0.390, and 0.687 l/cm^2^, respectively, after 72 h from the treatment of *S. oryzae* (Table 2).

**Table 2:** Toxicity values of Sesame, Clove, and Cinnamon nanoemulsion with and without adjuvant against *S. oryzae* 72 h after exposure.

Formulation	LC_50_ µl /cm^2^	Confidence limits		LC_90_ µl /cm^2^	Confidence limits		Slope	X^2^
		Lower	Upper		Lower	Upper		
Sesame (NE)	0.071	0.056	0.088	0.687	0.384	2.245	1.30 ± 0.24	4.87
Sesame (NE) + ARGAL	0.055	0.023	0.073	0.213	0.220	1.255	2.19 ± 0.25	10.87
Sesame (NE) + Atlox 4838B	0.032	0.019	0.091	0.138	0.044	3.105	2.00 ± 0.26	13.9
Clove (NE)	0.052	0.039	0.064	0.390	0.258	0.837	1.47 ± 0.24	0.70
Clove (NE) + ARGAL	0.056	0.113	0.1719	0.326	0.526	0.816	1.67 ± 0.24	8.72
Clove (NE) + Atlox 4838B	0.022	0.013	0.172	0.111	0.026	0.806	1.81 ± 0.29	14.9
Cinnamon (NE)	0.064	0.054	0.074	0.295	0.222	0.465	1.94 ± 0.24	5.823
Cinnamon (NE) + ARGAL	0.031	0.065	0.155	0.130	0.225	0.274	2.05 ± 0.26	13.3
Cinnamon (NE) + Atlox 4838B	0.035	0.0157	0.043	0.112	0.088	0.217	2.57 ± 0.26	8.67

**Fig. 5:**
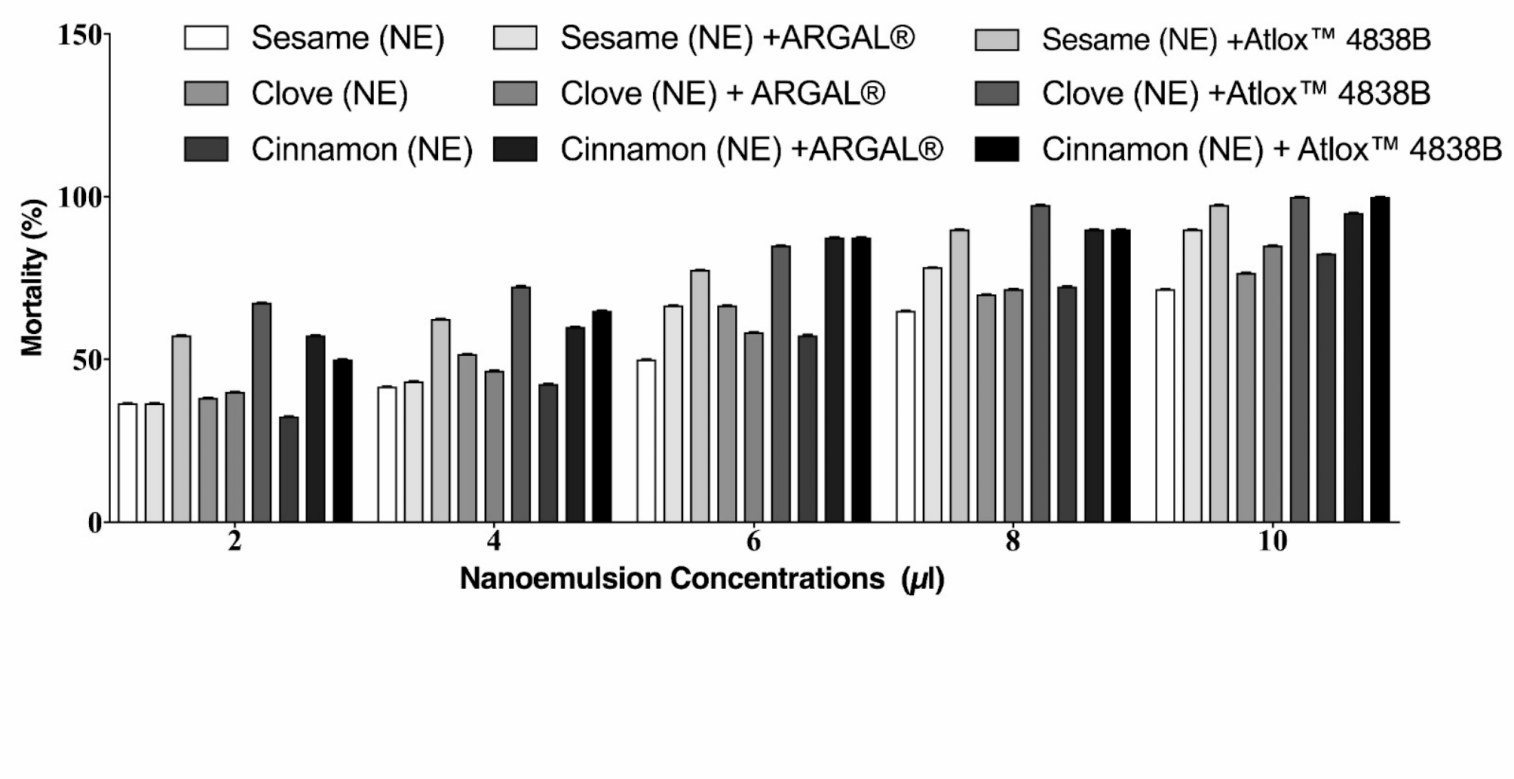
The impact of adding adjuvants to clove, cinnamon, and sesame nanoemulsion on the *S. oryzae* mortality rate after 72 h of exposure. Bar charts are generated using data from four biological replications with two technical replicates per replication. Each bar represents the mean, and the error bar indicates the standard deviation (± SD). This figure was drawn with GraphPad Prism 8 (9.4.1, (458) Serial number: GPS-2567891-8A130A8A228).

The results as shown in Fig. 5, 10µl treatment of Clove, Cinnamon, and Sesame nanoemulsion with Atlox 4838B adjuvant has higher mortality rates of *S. oryzae*, followed by Cinnamon, Sesame, and Clove nanoemulsion with ARGAL adjuvant after the 72 h of contact test than those of Cinnamon, Clove, and Sesame nanoemulsion alone in 10µl treatment. The mortality percentages of Clove, Cinnamon, and Sesame nanoemulsion with Atlox 4838B were 100, 100, and 97.5%, respectively, in 10 µl of treatment. The mortality rates were significantly higher in the 10µl treatment of Clove, Cinnamon, and Sesame nanoemulsion with adjuvant compared with the nanoemulsion without adjuvant. Thus, there is an increase in *S. oryzae* mortality percentages by adding adjuvants to the nanoemulsion formulation.

## Discussion

During the current study, after preparation, the nanoemulsion formulation for sesame, cinnamon, and clove oils with and without adjuvant was characterized. Droplet size and its distribution were measured by dynamic light scattering (DLS), which plays a significant role in emulsion stability^51,52^. The tiny size of nanoemulsion is desirable for maximum efficiency. A full picture of the droplet size population and distribution in sesame (NE) and cinnamon (NE) formulations was obtained by analyzing dynamic-light-scattering (DLS) data. The average droplet size recorded the lowest value of droplet diameter size (34.09 and 37.8 nm, respectively) compared to NEs Clove (NE) was 42.5 nm. While the particle size of the sesame, cinnamon, and clove formulations with adjuvant recorded a slight increase in droplet size compared to the same formulation without adjuvant, and this hypothesis was further supported by^29,53,54^. Generally, all studied formulations in the range of 20 to 200 nm corresponded to the typical droplet size of O/W nanoemulsion^55,56^. Smaller droplet sizes in nanoemulsions will result in a stable formulation by enhancing Brownian motion and decreasing attractive forces^57,58^.

PDI shows how homogeneous (in size) the droplets are in the formulation. It is a unitless value that ranges from 0 to 1^59^; the lower the value, the more homogenous the sample. PDI values close to one indicate a heterogeneous or multimodal distribution, while values below 0.2 indicate an extremely narrow dispersion of nanoparticles and uniformity among oil droplet sizes or monomodal distributions and, consequently, better stability^60^. In the present study, values recorded 0.04, 0.07, and 0.05 for sesame, clove, and cinnamon, respectively; it almost monodisperses. While adding adjuvant to the nanoemulsion formulation, it recorded a slight increase in PDI than without adjuvant to become a midrange. A sample is almost monodispersed if the PDI is less than 0.08 and the midrange is between 0.08 and 0.70. The distribution algorithms perform optimally within this range^61^. A PDI around 1 (> 0.7) denotes a wide droplet size dispersion^62,63^.

ARGAL and Atlox 4838B adjuvants were used to optimize the viscosity of Sesame, Clove, and Cinnamon nanoemulsions, resulting in a stable formulation. Current findings are in accordance with the report of^64^ stated that the viscosity of the nanoemulsion formulation is around 27 mPa-s, which makes it ideal for spreading and spraying on crops. Also,^28^ demonstrated that neem NE combined with a botanical adjuvant can provide better adherence on applied surfaces for good bio-efficacy and long-term stability during storage. The current results indicated that if the formulation’s surface tension decreases, the area of contact will increase, in addation, caused increase the area of contact between the sprayed surface and the spray and improving the wetting and spreading properties. These justifications concur with the findings of^32^. Moreover^65^, suggest that it could result in a rise in pesticidal effectiveness. Like that observed in^28^ reported that low surface tension in neem NE with adjuvant allows for improved formulation spreading on brinjal leaves with high bio-efficacy in comparison to neem NE without adjuvant^30,37,66^.

Zeta potential is a crucial factor that directly influences the stability of nanoemulsions^67^. It gives information regarding the homogeneous behavior of the nanoemulsion. Particles having zeta potentials greater Values above ± 30 mV are regarded as stable. Because the electric charge of the droplets is large, it is reasonable to suppose that repulsive interactions between droplets exist in the nanoemulsion. A negative zeta potential value in a dispersed emulsion inhibits coagulation and coalescence by establishing repulsive forces between droplets that are greater than their attraction forces^68^. The findings showed that sesame, clove, and cinnamon nanoemulsions with adjuvant increased the value of zeta potential, then alone. Furthermore, all the zeta potentials are in the negative zone, indicating that the formulation is stable. A negative zeta potential value creates repulsive forces higher than attraction forces among droplets, preventing coagulation and coalescence in dispersion emulsion. The zeta potential measures the surface charge of the emulsion droplet, showing the physical stability of nanoformulations^69^. Zeta potential provides electrophoresis mobility data that is relevant to the stability of the NE formulation. A greater value suggests better nanoformulation stability due to increased droplet repulsion, which prevents coagulation and flocculation^70^. Based on SEM images, all investigated nanoemulsion formulations had spherical or quasi-spherical morphologies. The surface morphology and shape of all investigated nanoemulsion formulations appeared spherical or quasi-spherical, uniform, and monodispersed morphologies, and the sizes of the nanodroplets corresponded to the ones measured with DLS. Spherical forms are commonly observed in nanoemulsion pesticides^71,72^. The spherical shape and absence of aggregation indicate the physical stability of the nanoemulsion. This stability may be attributed to the presence of surfactant and adjuvant. In contrast, Sesame (NE) + Atlox 4838B have a minor aggregation in the SEM image and is attributable to the sample drying process before the analysis; this is a common characteristic of emulsion systems in SEM analysis^73,74^. However, droplet sizes in SEM images corresponded well with those assessed by DLS. These findings are consistent with^75,76^, who used SEM to study the surface morphology of nanoemulsions and documented their spherical shapes. Abd El Salam et al.^77^ demonstrated that studies using transmission electron microscopy showed that the nanoemulsions were effectively generated at the nanoscale and mostly displayed a spherical shape. Also^78^, stated that the TEM examination clearly demonstrated that the nanoemulsions loaded with colistin-niclosamide were spherical, non-adhesive, non-aggregating, and equally dispersed in space; the diameters of the nanodroplets matched to those measured with DLS. The viscosity of the studied nanoemulsions without adjuvant was found to be less viscous than the tested nanoemulsions with adjuvants. The viscosity value can be influenced by the type of surfactants, organic phase constituents, and oil viscosity. Pesticide nanoemulsion has a low viscosity since it is classified as O/W with significant water loading. Nevertheless, the viscosity of nanoemulsion can be modified by surfactant concentration^79^.

Surface tension of the developed sesame, clove, and cinnamon NE formulations by adding the adjuvant reduced the surface tension. When the surface tension of a formulation reduces, the area of contact is higher, causing an increase in the area of contact between the sprayed surface and the spray, as well as enhancing the wetting and spreading capabilities^80^. During this research the influence of adjuvants on Sesame, Clove, and Cinnamon nanoemulsions stability was detected. Stability is a critical characteristic in nanoemulsion systems. Nanoemulsions’ tiny droplet size gives them stability against creaming or sedimentation due to Brownian motion, and as a result, the diffusion rate is higher than the sedimentation rate generated by gravity force (see, Table 1). Comparable outcomes closely align with the conclusions of^55,81,82^**.** During the current experiment the measurements of the zeta potential showed that the nanoemulsion is homogenous. Also, these data illustrated that zeta potentials for Sesame, Clove, and Cinnamon nanoemulsions with and without adjuvants within the zone of negative zeta potential. The results demonstrated the stability of the sesame, clove, and cinnamon nanoemulsions because of the constant distribution and varying values of the nanoformulation with adjuvant. These findings concur with a prior study by^83,84^**.**

The pH value acts as a stabilizing indicator for nanoemulsions, depending on the surface properties of the droplet. In our finding, it was observed that the pH of all tested formulations with and without adjuvant exhibited an acidic to slightly acidic pH except sesame (NE) with Atlox 4838B exhibited neutral pH. In the current investigation, the adjuvant was shown to slightly enhance the pH value and stabilize it, hence reducing the degradation of the tested formulation. The obtained results were confirmed by 28 who found that using a botanical adjuvant stabilizes the pH to slightly acidic to prevent neem NE from degrading while being stored. Previous research has shown that excessive alkalinity or acidity in nanoformulations degrades the active component, reducing the formulation’s bioefficacy^85^. In another study, it was found that changing pH aggregates and destabilizes nanoemulsions during storage^86^. Abd El Salam et al.^77^ found that frankincense, turmeric, and sesame NE formulations had a slightly acidic pH, which is typical of stable nanoemulsions.

The insecticidal activity of enhancement clove, cinnamon, and sesame NE against rice weevils, *S. oryzae*, as shown in the results that adding Calcium Alkyl Benzene Sulphonate (Atlox 4838B) adjuvant to clove, cinnamon, and sesame NE performed higher toxicity against the rice weevils, *S. oryzae*, after a 27-h exposure period than other formulations. Also in the mortality rate, the results of 10µl treatment of Clove, Cinnamon, and Sesame NE with Atlox 4838B adjuvant have higher mortality rates, followed by Cinnamon, Sesame, and Clove NE with ARGAL adjuvant after the 72 h of contact test than those of Cinnamon, Clove, and Sesame NE alone in 10µl treatment. However, the above-mentioned results conform with the findings of a lot of scientists on various nano formulations and adjuvants; furthermore^28^, reported that neem nano-emulsion with botanical adjuvant provided long-term storage stability as well as better adhesiveness and wetting with lower surface tension, as well as neem NE with adjuvant showed remarkable insecticidal effectiveness against whitefly. Furthermore^29^, found that adding adjuvants to olibanum NE improved stability and lowered contact angle and surface tension more effectively than olibanum NE alone. Additionally, the olibanum NE, with or without adjuvants, was the most effective chemical against the 2nd instar larvae of the spiny bollworm Earias insulana. Furthermore^30^, demonstrated that the solid lipid nanoparticles (SLNs) of citronella oil combined with three adjuvants increased the toxicity and mortality percentage of Spodoptera littoralis larvae, as well as stability, more than the nanoformulation alone. Also, chemical pesticides such as abamectin, lambda-cyhalothrin, imidaclopride, emamectin benzoate, and oxamyl were shown to be more effective and to have longer-lasting effects when PEG 600 di-oleate adjuvants were added^32^. Adjuvants improved the efficacy of lambda-cyhalothrin formulations, according to^34^. Consequently, the number of treatments each season and the pace at which pesticides are applied can both be decreased by using adjuvants.

## Conclusions

The current research focused on using nanoemulsions to create lipophilic active-loaded products for pesticide application to develop a green efficient, and stable pesticide formulation. During this experiment, certain nano emulsions nemaly, Calcium Alkyl Benzene Sulphonate (Atlox 4838B), and non-ionic surfactant based on trisiloxane ethoxylate (ARGAL), were tested against *S. oryzae*. Current results showed that all formulation were penal, achieving nanometric size for all compounds. The bioassay test (LC_50_) values for *S. oryzae* adults indicated that Clove, Sesame, and Cinnamon nano emulsions with Atolx adjuvants were the most effective under laboratory conditions, where the LC_50_ values were 0.022, 0.032 and 0.035 µL/cm^2^ respectively after 27 h. Clove, Cinnamon, and Sesame nanoemulsion (NE) with 0.01% (w/w) adjuvant exhibited remarkable insecticidal activity against *S. oryzae* L., of 100, 100 and 97.5% respectively by in vitro assay. These two categories of adjuvants enhanced the spray performance, maximum retention, enhanced bioavailability, and control impact by improving the viscosity, surface tension, and water dispersibility. The suspension that was created with Atlox 4838B outperformed the others in terms of wettability, surface tension, and *S. oryzae* control. Dynamic light scattering (DLS) revealed that the particle size was nanometrically created, whereas SEM revealed that the studied NE formulations, both with and without adjuvants, primarily had spherical or quasi-spherical morphologies. The particles ranged in width from 20 to 100 nm. Whereas for NE, a polydispersity was almost monodispersed. While the NE formulation becomes a midrange when an adjuvant is added. Using the exception of sesame (NE) using AtloxTM 4838B, which showed neutral, the pH ranged from 4.27 to 6.96, suggesting an acidic to mildly acidic pH. Compared to the studied nanoemulsions with adjuvants, the viscosity of the nanoemulsions without adjuvants was lower. There is no phase separation, creaming, or crystallization in the kinetically stable nanoemulsion. For sesame, clove, and cinnamon nanoemulsions with adjuvant, a negative zeta potential value raised the value of zeta potential compared to when used alone. Additionally, the adjuvant decreased the NE formulations’ surface tension.

## Data Availability

The datasets generated or analyzed during the current study are available on reasonable request by the Corresponding author(s): mazennour2@yahoo.com (M.I.M).

## Abbreviations

°C: Degree Celsius
LC50: Lethal concentrations, which cause 50% mortality in the population
L.S.D: Least significant different
*S. oryzae*: *Sitophilus oryzae* L.
NE: Nanoemulsion
PI: Polydispersity index
